# *In silico* Prediction of Human Secretory Proteins in Plasma Based on Discrete Firefly Optimization and Application to Cancer Biomarkers Identification

**DOI:** 10.3389/fgene.2019.00542

**Published:** 2019-06-06

**Authors:** Jian Zhang, Yu Zhang, Zhiqiang Ma

**Affiliations:** ^1^School of Computer and Information Technology, Xinyang Normal University, Xinyang, China; ^2^Henan Key Laboratory of Education Big Data Analysis and Application, Xinyang, China; ^3^Information Engineering College, Huanghuai University, Zhumadian, China; ^4^Henan Key Laboratory of Smart Lighting, Zhumadian, China; ^5^Department of Computer Science, College of Humanities & Sciences of Northeast Normal University, Changchun, China

**Keywords:** secretory proteins, human plasma, human proteome, cancer biomarker, discrete firefly algorithm

## Abstract

The early control and prevention of cancer contributes effectively interventions and cancer therapies. Secretory protein, one of the richest biomarkers, is proved important as molecular signposts of the physiological state of a cell. In this work, we aim to propose a proteomic high-throughput technology platform to facilitate detection of early cancer by means of biomarkers that secreted into the bloodstream. We compile a new benchmark dataset of human secretory proteins in plasma. A series of sequence-derived features, which have been proved involved in the structure and function of the secretory proteins, are collected to mathematically encode these proteins. Considering the influence of potential irrelevant or redundant features, we introduce discrete firefly optimization algorithm to perform feature selection. We evaluate and compare the proposed method SCRIP (Secretory proteins in plasma) with state-of-the-art approaches on benchmark datasets and independent testing datasets. SCRIP achieves the average AUC values of 0.876 and 0.844 in five-fold the cross-validation and independent test, respectively. Besides that, we also test SCRIP on proteins in four types of cancer tissues and successfully detect 66∼77% potential cancer biomarkers.

## Introduction

Cancer is a major public health problem in the world, a recent survey reports that more than 1.7 million new cancer cases were diagnosed in the United States in 2018 (Siegel et al., 2019). The number is even three times higher than that in China. Early detection of cancer facilitates timely diagnosis and therapy in its pre-invasive state prior to metastasis, which increases the chances of successful treatment (Kessler, 2017). For instance, the cancers of the breast, larynx, colon and skin can be effectively controlled in their early state. As a result, they can benefit from early prevision and diagnosis (Medicine et al., 2003). Recently, increasing efforts and financial resources are invested in early cancer detection research. Among these efforts, blood assays detecting promises high probabilities on patients’ survival for early cancers (Kim et al., 2013). Additionally, blood assays detecting is non-invasive and financially reasonable (El-Zein et al., 2017), which makes it widely available.

As one of the rich source of biomarkers, secretory proteins are favored by biologists because they show various states of the cells at real time under given conditions (Tonry et al., 2016). Featured by the capability of reflecting various stages of diseases, secretory proteins are desirable for diagnosis, prognosis, etc., Particularly, in clinical diagnosis, direct analysis of blood/plasma is widely used as one of the non-invasive patient screenings (Lin et al., 2011). By coincidence, the proteins secreted by cells as responses to various stimuli are most likely secreted into blood/plasma. As a result, the accurate recognition of secretory proteins as potential cancer biomarkers is becoming a promising approach.

Compared with time-consuming and labor-intensive biochemical or biophysical approaches, computation-based methods are becoming more and more popular in high-throughput *in-vivo* research. Benefit from their convenience and effectiveness, biologists focus on the *in silico* research to handle the explosive growth of unknown protein sequences. Hung et al. used informative physicochemical properties together with inheritable bi-objective genetic algorithm to predict secretory proteins (Hung et al., 2010). Liu et al. adopted manifold ranking algorithm and regarded this problem as a semi-supervised problem (Liu et al., 2010). SecretP was designed for distinguishing three types of proteins (classically secreted proteins, non-secreted proteins, and non-classically secreted proteins) in mammals. It also fusing several new features into Chou’s pseudo-amino acid composition (Yu et al., 2010). Hong et al. collected features between proteins found in the normal urine and that deemed not to be urine excretory. They trained the model and used it for the identification of gastric cancer markers in urine (Hong et al., 2011). NClassG+ was a classifier that designed for non-classically secreted gram-positive bacterial proteins (Restrepo-Montoya, 2011). Luo et al. (2012) used PSSM together with auto covariance scheme. The former represented the multiple sequence alignment profiles, and the latter was applied to take the neighboring effects of the sequences into account. Wang et al. proposed a sequence-based method for identification of human salivary proteins from blood circulation. They also used the model and predicted 31 candidate biomarker proteins in saliva for breast cancer (Wang et al., 2013). Sun et al. proposed a mathematical method to predict saliva-secretory proteins. Using the predictor, they predicted potential salivary biomarkers for head and neck squamous cell carcinoma (Sun et al., 2015). iMSP was a sequence-based predictor for identification of mammalian secreted proteins (Zhang et al., 2018). It also predicted 272 potential secreted proteins with high confidence.

The above-mentioned research contributes to the knowledge of secretory proteins. However, as far as we know, there exists several shortcomings, which should be further considered. First, few research investigates the intrinsic attributes of secretory proteins. Some significant properties of secretory proteins have remained unknown; second, for a typical machine learning approach, feature selection is not only necessary but also crucial for constructing a robust model (Zeng et al., 2017). The existences of potential redundant or noisy features will somewhat influence the feature space as well as ruin the constructed model; third, secretory proteins find wide application in early cancer detection research. Up to now, no specific predictor is proposed for the *in silico* identification these special secretory proteins that serve as cancer biomarkers.

To successfully address the above-mentioned issues, we focus on the challenge of proposing an accurate predictor for the identification of human secretory proteins in plasma/blood. A number of sequence-based features that related to secretory proteins are used to encode the proteins. We perform comprehensive computation-based analysis and statistics for these proteins. Considering the fact that machine learning strategy is sensitive to the choice of feature space, we introduce discrete firefly optimization algorithm to perform feature selection. To further test the generalization of the proposed method, we perform both benchmark and independent test and compare SCRIP with current predictors. Besides that, we also use SCRIP to recognize potential secretory proteins that serve as biomarkers on four different types of cancers. SCRIP is expected to become a promising tool for predicting and analyzing human secretory proteins in plasma/blood.

## Materials and Methods

### Framework of the Proposed Method

**FIGURE 1:**
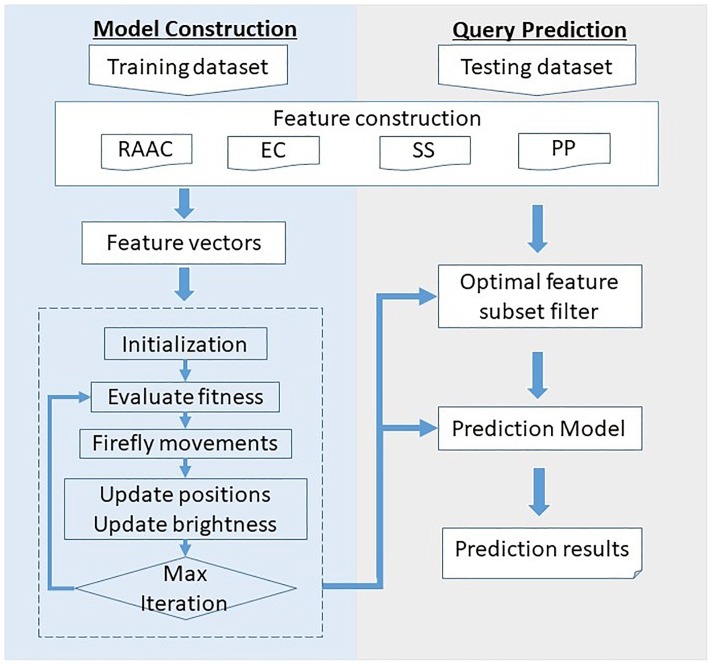
Overall framework of the proposed SCRIP method.

Figure 1 illustrates the framework of the proposed SCRIP. The overall framework is consists of two parts, namely model construction and query prediction. For the model construction part, the training dataset is first quantified into various type of features. Then, the feature space is filtered for optimal feature subset by adopting discrete firefly optimization algorithm. Next, the optimal feature subset is fed into the logistic regression to generate the training model. For the query prediction part, the query protein is encoded as a feature vector, and then filtered by the optimal feature subset. After predicting by the pre-trained model, it outputs the probability of being a secretory protein.

### Benchmark Datasets

We collect a total number of 20,325 human proteins from Swiss-Prot (December 16, 2018). From these human proteins, we further collect 505 secretory proteins. To evaluate whether these secretory protein has been detected experimentally in blood/plasma, we check these proteins against Human Plasma Proteome Project (Schwenk et al., 2017). We remove the proteins that have less than 50 residues because these proteins are tend to be segments. Then, blastclust (Altschul et al., 1997) is used to cluster these proteins with the threshold of 30%. For each cluster, we randomly pick one protein as the representative. Finally, we obtain 332 secretory proteins in plasma/blood as positive samples. To compile negative samples, we collect the rest 14,368 human proteins which are not annotated as secretory proteins.

We randomly pick 250 secretory proteins from positive dataset and 250 non-secretory proteins from negative dataset as the training dataset. The rest 82 secretory proteins and randomly picked 82 non-secretory proteins are used as the testing dataset. To avoid potential bias on the sampling, we repeat this procedure for ten times. The reported results are the average and standard deviation of the ten experiments. For comparison with previous studies, we also adopt the independent testing datasets from iMSP (Zhang et al., 2018). The datasets that used to generate and evaluate SCRIP are free available at http://www.inforstation.com/webservers/SCRIP/.

### Feature Construction

#### The Features of Relative Amino Acid Composition

As the basic element of proteins, amino acids play vital roles in determining the protein folding into a specific structure. However, all residues in a protein are not equally important. Some are essential for constructing stable structure and function of the proteins, while others can be readily replaced. The sequence of amino acids determines protein’s activity and function. Given the importance of the amino acids, the features of amino acid composition has been widely used in mathematically describing protein structures and functions (Wei et al., 2017; Zhang et al., 2017a).

The features of amino acid composition is quantified as relative differences between secretory proteins and non-secretory proteins. We compute relative amino acid composition (RAAC) using Composition Profiler (Vacic et al., 2007). Specifically, we calculate RAAC for secretory proteins against Swiss-Prot database and secretory proteins against non-secretory proteins.

#### The Features of Secondary Structure

The secondary structure involves protein tertiary structure and function sites. The proteins, which are enriched with folds, are usually in a stable arrangement. Although molecular evolution in families of related proteins tend to form similar structures, it may eliminate some similarities on sequence or peptide motifs (Wink, 2018). This gives rise to the proteins belong to the same families with similar secondary structures because they all diverge from a common ancestor.

Besides secondary structure elements, some proteins show propensities on certain super-secondary structure motifs (Koch and Schaefer, 2018). For instance, “β-α-β” is a typical common super-secondary structure. The central α helix connects the C-termini of the first β strand, and the N-termini of the second β strand. This results packing its side chains against the β strand and therefore shielding the hydrophobic residues of the β strands from the surface (MacCarthy and Perry, 2019). Here, we introduce the features of secondary structure (SS), which contains both putative secondary structure probabilities as well as local super-secondary structure motifs.

#### The Features of Evolutionary Conservation

With the evolution of generations, mutation occurs and randomly changes residues in any positions of proteins (Zou et al., 2015). Evolutionary conservation indicates that a set of residues or peptide has been maintained by natural selection. The conserved parts of a sequence are always related to its space skeleton or biological function (Zou et al., 2018). In this study, evolutionary conservation is calculated by aligning the protein primary sequence against Swiss-Prot database. We use psi-blast (Altschul et al., 1997) to perform the sequence alignment and obtain the position-specific scoring matrix (PSSM) as follows:

(1) $$\text{PSSM}=\left[\begin{array}{ccc} S_{1\to A}\;S_{2\to R} & \cdots & S_{1\to V}\\ S_{2\to A}\;S_{2\to R} & \cdots & S_{2\to V}\\ \vdots \;\vdots & \cdots & \vdots \\ S_{\text{L}\to \text{A}}\;S_{\text{L}\to \text{R}} & \cdots & S_{\text{L}\to \text{V}} \end{array}\right]$$

*S*_i→AA_ quantifies the probability of the *i*-th amino acids (AA) being substituted by AA during the evolutionary process. A higher score indicates this substitution is favored, while the lower value represents the opposite. The preferences of 20 amino acids being substituted are statistically classified and analyzed by using the following formula:

(2) $$S_{\text{i,j}}=\sum_{\text{i}=1}^{L}S_{\text{i}\to \text{j}}\times \delta \{\begin{array}{cc} \delta =1, & R_{\text{i}}={\mathrm{AA}}_{\text{j}}\\ \delta =0, & R_{\text{i}}\neq {\mathrm{AA}}_{\text{j}} \end{array}$$

where *R*_i_ indicates the *i*-th residues in the protein sequence. The *S*_i,j_ is further normalized by using logistic function to eliminate the influences of the length of the proteins. The features of evolutionary conservation (EC) for each sequence is encoded as a vector with 400 dimensions.

#### The Features of Physicochemical Properties

We collect eleven common used physicochemical properties (PP) to encode secretory proteins. These properties include aliphatic (Avrahami et al., 2001), sulfur (Suliman et al., 2002), aromatic (Scheiner et al., 2002), hydrophobic (Strub et al., 2004), charge (Heard and Weiner, 1998), polar (Kamtekar et al., 2010), positive (Heard and Weiner, 1998), acidic (Goodwin et al., 2010), small (Sghaier et al., 2013), tiny (Sghaier et al., 2013), and hydroxylic (Chai and Zhang, 2018). For each properties, we first sum up the values for each of the residues in the whole sequence, and then calculate the average values for each properties.

### Logistic Regression

In this work, we utilize logistic regression to build the models. Logistic regression is a simple non-linear regression. Consider its simplicity and effectiveness, logistic regression has been recently widely used in predicting protein structures and functions (Zhang et al., 2017b). Logistic regression assigns various weights to each features in the optimal feature subsets. It is easy to identify the valuable features and further investigate the reasons. This leaves informative clues for future researchers. Moreover, the outputs of logistic regression is between zero and one, which indicates the probability of a query protein to be non-secretory protein (0%) or secretory protein (100%). Since logistic regression is a simple non-linear regression, it has less chance to lead to overfitting. This attribute endows it a good generalization. Particularly, the effectiveness of logistic regression also promises the large scale of application, such as human proteome.

#### Discrete Firefly Optimization Algorithm

Discrete firefly optimization algorithm is proved to be a powerful nature-inspired algorithm for solving complex discrete problems, such as flow shop scheduling (Marichelvam et al., 2014), fault diagnosis (Fister et al., 2013), and feature selection (Long et al., 2017). Discrete firefly optimization algorithm follows three basic rules (Fister et al., 2013). First, a firefly will be attracted by other fireflies regardless their sex; second, attractiveness is proportional to their brightness and decreases with the distance among them increases; third, the landscape of the objective function determines the brightness of a firefly.

In the standard firefly algorithm, the light intensity I of a firefly is defined as follows:

(3) $$I(r)=I_{0}e^{-\gamma r^{2}}$$

where *I*_0_ denotes the light intensity of the source. Light absorption is approximated using the fixed light absorption coefficient γ. The distance between any two fireflies is expressed as:

(4) $$r_{\text{i,j}}=\left\|s_{\text{i}}-s_{\text{j}}\right\|=\sqrt{\sum_{k=1}^{\text{k}=\text{n}}{\left(s_{\text{ik}}-s_{\text{jk}}\right)}^{2}}$$

where *n* is the dimensionality of the problem. The movement of the *i*-th firefly is attracted by another more attractive firefly *j*, and is applied as:

(5) $$s_{\text{i}}=s_{\text{i}}+\beta_{0}e^{-\gamma r_{\text{ij}}^{2}}\left(s_{\text{i}}-s_{\text{j}}\right)+\alpha \in_{\text{i}}$$

where _i_ is a random number drawn from Gaussian distribution. In this work, the position of a firefly is changed from binary bits to real values by using sigmoid function:

(6) $$s_{\text{i}}=\frac{1}{1+e^{-x_{\text{ik}}}}$$

In this study, the fitness function is consist of two parts, namely the prediction accuracy (MCC) as well as the number of selected features. Thus, we defined the fitness as:

(7) $$f_{i}=\omega_{\alpha }{\mathrm{MCC}}_{i}+\omega_{\beta }(1-\frac{n}{N})i$$

where ω_α_ and ω_β_ are the weights of the predictive accuracy of the model and the size of optimal feature space, and ω_α_ + ω_β_ = 1.

### Evaluation Criteria

The proposed predictor outputs both binary and real-valued predictions. To compare with previous methods, we introduce both binary and real-valued predictions criteria. In detail, binary predictions, namely secretory proteins vs. non-secretory proteins, are evaluated using sensitivity, specificity, precision, accuracy, F1-measure (F1), and Matthews correlation coefficient (MCC). Equation 8∼13 give the definition of these criteria. True positives (TPs) and true negatives (TNs) stand for correctly recognized secretory and non-secretory proteins, respectively. False positives (FPs) indicate incorrectly predicted non-secretory proteins as secretory ones, while false negatives (FNs) represent incorrectly predicted secretory proteins as non-secretory ones.

(8) $$\mathrm{Sensitivity}=\frac{\mathrm{TP}}{\mathrm{TP}+\mathrm{FN}}$$

(9) $$\mathrm{Specificity}=\frac{\mathrm{TN}}{\mathrm{TN}+\mathrm{FP}}$$

(10) $$\mathrm{Precision}=\frac{\mathrm{TP}}{\mathrm{TP}+\mathrm{FP}}$$

(11) $$\mathrm{Accuracy}=\frac{\mathrm{TP}+\mathrm{TN}}{\mathrm{TP}+\mathrm{FP}+\mathrm{TN}+\mathrm{FP}}$$

(12) $$F1-\mathrm{measure}=2\times \frac{\mathrm{Sensitivity}\times \mathrm{Precision}}{Sensitivity+\mathrm{Precision}}$$

(13) $$\frac{\mathrm{TP}\times \mathrm{TN}-\mathrm{FN}\times \mathrm{FP}}{\sqrt{\left(\mathrm{TP}+\mathrm{FN})\times (\mathrm{TP}+\mathrm{FP})\times (\mathrm{TN}+\mathrm{FP})\times (\mathrm{TN}+\mathrm{FN}\right)}}$$

Binary prediction criteria may suffer from the imbalanced data, which benefits from the real-valued prediction criteria. The latter criteria is capable of providing unbiased assessment without considering the threshold. The research illustrates receiver operating characteristic curve (ROC curve) to demonstrate the overall predictive quantity. ROC curve plots the TPR (true positive rate) against the FPR (false positive rate) at various thresholds. Besides that, we also calculate the area under ROC curve (AUC).

## Results and Discussion

### The Characteristics of the Considered Features

In this work, we encode the proteins by calculating the two types of RAAC values. That is, the RAAC of secretory proteins against Swiss-Prot, and the secretory proteins against non-secretory proteins. As listed in Table 1, compared with the amino acid distribution in Swiss-Prot, secretory proteins prefer cysteine, threonine, serine, proline. When compared with non-secretory proteins, secretory proteins are significantly enriched in threonine, while depleted in lysine, glutamine and arginine.

**Table 1:** The RAAC values for secretory proteins against Swiss-Prot database and secretory proteins against non-secretory proteins.

AA type	Secretory proteins vs. Swiss-Prot	Secretory proteins vs. non-secretory proteins	AA type	Secretory proteins vs. Swiss-Prot	Secretory proteins vs. non-secretory proteins
A	**−0.138 (0.000)**	**−**0.042 (0.000)	M	**−0.125 (0.000)**	0.003 (0.766)
C	**0.578 (0.000)**	0.098 (0.000)	N	**−**0.088 (0.000)	0.067 (0.000)
D	**−**0.085 (0.000)	0.020 (0.051)	P	**0.274 (0.000)**	**−**0.057 (0.000)
E	**−**0.012 (0.073)	**−**0.098 (0.000)	Q	0.088 (0.000)	**−0.118 (0.000)**
F	**−**0.072 (0.000)	0.056 (0.000)	R	**−**0.086 (0.000)	**−0.136 (0.000)**
G	**−**0.063 (0.000)	0.002 (0.883)	S	**0.243 (0.000)**	**−**0.002 (0.537)
H	0.051 (0.000)	**−**0.068 (0.000)	T	**0.355 (0.000)**	**0.383 (0.000)**
I	**−0.225 (0.000)**	0.096 (0.000)	V	**−**0.045 (0.000)	0.090 (0.000)
K	**−0.128 (0.000)**	**−0.102 (0.000)**	W	**0.133 (0.000)**	0.068 (0.001)
L	**−**0.004 (0.319)	**−**0.038 (0.000)	Y	**−0.129 (0.000)**	0.042 (0.003)

*The values in brackets are the p-values. The bold indicates the significantly enriched (>0.1, p-value < 0.05) or depleted (<**−**0.1, p-value < 0.05) amino acid.*

**FIGURE 2:**
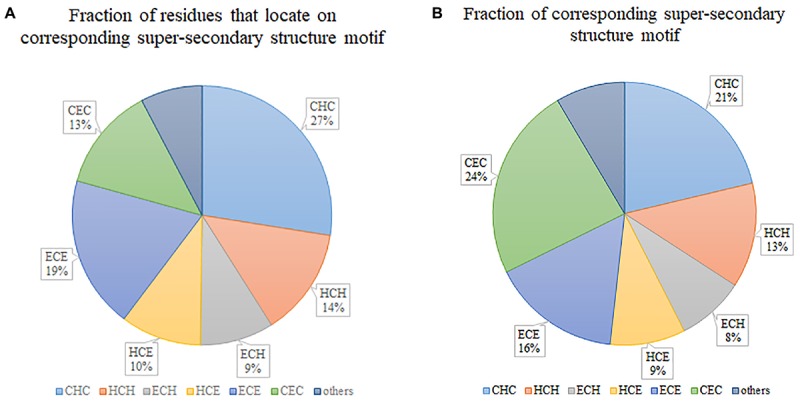
Statistics of super-secondary structure motifs in secretory proteins. **(A)** Fraction of residues that locate on corresponding super-secondary structure motif. **(B)** Fraction of corresponding super-secondary structure motif.

Figure 2A illustrates the fraction of residues that locate on various super-secondary structure motifs. “CHC” occupies the biggest 27%, which means that most of the residues tend to locate on this type of motif. However, if we consider the fraction of super-secondary structure motifs, “CHC” is not the biggest. By contrast, “CEC” occupies the largest part (Figure 2B), although only 13% of residues locate on it. This indicates that the length of “CEC” is about half of “CHC” in secretory proteins. Generally, six prevalent super-secondary motifs occupy the majority of all considered motifs. They are “CHC,” “HCH,” “ECH,” “HCE,” “ECE,” and “CEC,” respectively.

**FIGURE 3:**
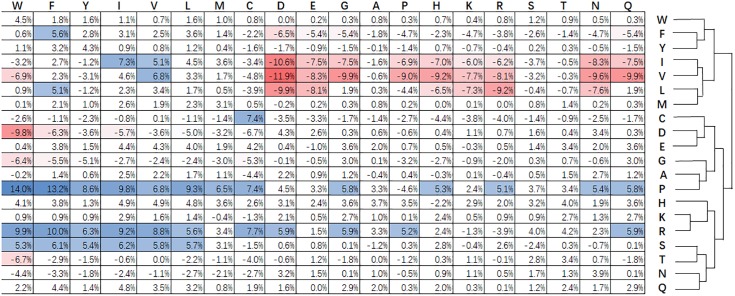
Relative difference of evolutionary conservation matrix between secretory and non-secretory proteins. The 20 amino acids residues are shown at the top and right. Values higher than 5% indicate the corresponding substitution is favored by secretory proteins compared with that of non-secretory proteins, and are colored blue. The red grids are the values lower than –5% and stand for the opposite. The amino acids are grouped using agglomerative clustering with complete linkage.

Figure 3 shows statistical frequencies of residues in public available super-secondary structure motifs. Compared with non-secretory proteins, secretory proteins tend to favor R-X, P-X, Q-X, E-X, H-X, and W-X related substitutions. Particularly, R-X, P-X and S-X related substitutions are most favored. By contrast, V-X, I-X, and L-X related substitutions are not enriched. C-X, i.e., cysteine-rich secretory proteins predominantly found in the mammalian male reproductive tract and in the venom of reptiles (Sevier and Kaiser, 2002). The formation of disulfide bonds contribute to the protein folding and stabilization of space structure (Sevier and Kaiser, 2002). This procedure will make proteins easily been secreted into the extracellular medium. Zhang et al. (2017c) pointed out that branched chain amino acids (isoleucine, leucine and valine) enhance protein synthesis and secretion. As a result, the substitutions for I-X, L-X, and V-X are relatively lower that the non-secretory proteins.

### The Performance of the Considered Features

In this section, we evaluate each type of features on the training dataset by using 5-fold cross-validation. We randomly pick 250 proteins secretory proteins and the equal number of non-secretory proteins. We repeat the under-sampling for ten times to avoid potential bias. Table 2 lists the average and stand deviation of the ten experiments. Generally, the considered four types of features all produce decent predictive results. RAAC gives out the average MCC of 0.196 and AUC of 0.645. SS produce the average MCC of 0.187 and the AUC of 0.647. Although we use eleven physicochemical properties, the constructed model still produce a decent average MCC of 0.224 and the AUC of 0.652. By contrast, EC yields the highest prediction performance with the average MCC of 0.375 and the AUC of 0.749.

**Table 2:** The predictive performance of different types of features on training dataset using 5-fold cross-validation.

Type of features		Sensitivity	Specificity	Precision	Accuracy	MCC	F1	AUC
RAAC	Average Stdev	0.623 ± 0.019	0.573 ± 0.015	0.593 ± 0.009	0.598 ± 0.010	0.196 ± 0.020	0.608 ± 0.012	0.645 ± 0.010
SS	Average Stdev	0.614 ± 0.013	0.573 ± 0.015	0.590 ± 0.011	0.593 ± 0.012	0.187 ± 0.023	0.601 ± 0.012	0.647 ± 0.019
EC	Average Stdev	0.684 ± 0.021	0.691 ± 0.018	0.689 ± 0.016	0.687 ± 0.016	0.375 ± 0.031	0.686 ± 0.017	0.749 ± 0.011
PP	Average Stdev	0.562 ± 0.018	0.660 ± 0.027	0.624 ± 0.023	0.611 ± 0.019	0.224 ± 0.039	0.591 ± 0.019	0.652 ± 0.011

*The threshold is set where the MCC achieve the maximum value.*

Next, we investigate that whether the combination of different types of features contributes the recognition of secretory proteins. The models that built on two types of features slightly increase the average AUC values when compared with that built on one type. This is also true for three types of features than that on two types of features (shown in Table 3). This indicates that each type of features contribute to the identification of secretory proteins. When using the considered four types of features, the model gives out the average AUC of 0.765, and the average MCC and F1 of 0.423 and 0.710, respectively.

**Table 3:** The predictive performance the combination of different types of features on training dataset using 5-fold cross-validation.

Type of features		Sensitivity	Specificity	Precision	Accuracy	MCC	Fl	AUC
RAAC+SS	Average stdev	0.630 ± 0.016	0.581 ± 0.015	0.601 ± 0.009	0.606 ± 0.009	0.212 ± 0.018	0.615 ± 0.010	0.647 ± 0.011
RAAC+EC	Average stdev	0.690 ± 0.020	0.698 ± 0.016	0.695 ± 0.014	0.694 ± 0.014	0.387 ± 0.027	0.692 ± 0.015	0.752 ± 0.009
RAAC+PP	Average stdev	0.625 ± 0.010	0.663 ± 0.019	0.650 ± 0.013	0.644 ± 0.010	0.289 ± 0.021	0.637 ± 0.009	0.656 ± 0.008
RAAC+SS+EC	Average stdev	0.692 ± 0.019	0.702 ± 0.018	0.699 ± 0.017	0.697 ± 0.017	0.394 ± 0.034	0.696 ± 0.017	0.652 ± 0.013
RAAC+SS+PP	Average stdev	0.645 ± 0.018	0.653 ± 0.009	0.650 ± 0.007	0.649 ± 0.009	0.298 ± 0.017	0.647 ± 0.012	0.651 ± 0.013
RAAC+EC+PP	Average stdev	0.698 ± 0.014	0.706 ± 0.012	0.703 ± 0.010	0.702 ± 0.010	0.404 ± 0.020	0.701 ± 0.011	0.757 ± 0.006
RAAC+SS+EC+PP	Average stdev	0.707 ± 0.017	0.716 ± 0.010	0.713 ± 0.008	0.711 ± 0.009	0.423 ± 0.018	0.710 ± 0.011	0.765 ± 0.010

*The threshold is set where the MCC achieve the maximum value.*

### Comparison of Different Feature Selection Approaches

We compare discrete firefly algorithm with other feature selection approaches. These approaches include LASSO (Least Absolute Selection and Shrinkage Operator) (Yamada et al., 2014) and two swarm optimization algorithms, namely particle swarm optimization and genetic algorithm. The initial parameters are set as follows: for LASSO algorithm, the lambda is set between 1 and 100, the predicted performance of the model with the highest AUC is kept. The corresponding feature subset is regarded as the optimal feature subset; for genetic algorithm, we set the crossover equals 0.6 and the mutation is 0.033; for discrete swam optimization algorithm, we set the C1 and C2 as 1 and 2, respectively; for discrete firefly algorithm, we set the randomness as 0.9 and the absorption coefficient as 0.5. Besides that, the populations/particles/fireflies for three algorithms are set as 50, and the max generation as 3000. We use the same fitness function to optimize the swarm optimization algorithms as well as the models. For Eq.7, we set *ω_α_* and *ω_β_* as 0.55 and 0.45, respectively. That is, we aim to select less number of features with the capability of produce high accuracy model.

**FIGURE 4:**
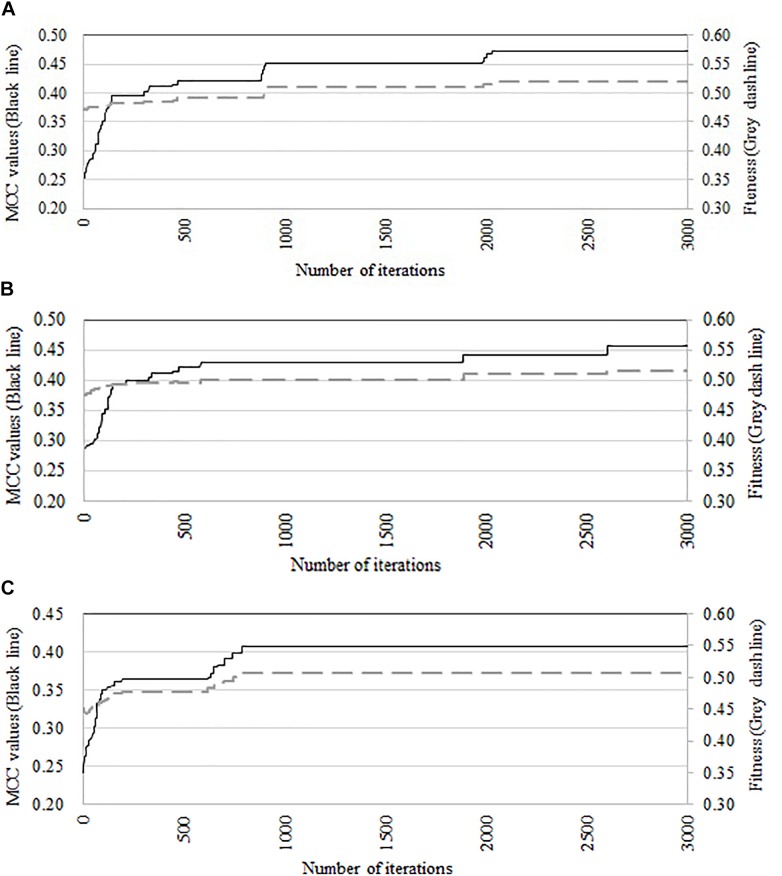
Comparison of predictive accuracy and fitness of three swarm optimization algorithms. **(A)** Discrete firefly algorithm, **(B)** discrete particle swam optimization, and **(C)** genetic algorithm.

We compare the performance of three optimization algorithms in feature selection as well as model construction (shown in Figure 4). With the increase of the iterations, the average fitness values of three considered algorithms all obviously rise. Table 4 lists the predictive performance of the considered four different algorithms. LASSO performs feature selection by quantifies the linear dependency between input features and output values (Fonti and Belitser, 2017). As listed in Table 4, LASSO gives out the average AUC of 0.784, and the MCC of 0.484, which is about 2.5% and 14.4% improvement than that of the direct combination of all features. The improvement is relative slight when compared with three swarm optimization algorithms. Concisely, the average MCC value for discrete firefly algorithm increases from about 0.28 to about 0.57. By contrast, discrete particle swam optimization and genetic algorithm produce the average MCC values from 0.28 to about 0.55, and 0.25 to 0.55, respectively. Moreover, we notice that, discrete firefly algorithm select 254 features. It is much less than that of discrete particle swam optimization (280 features) while slightly higher than genetic algorithm (233 features). Although LASSO selects the least number of features (74 for LASSO vs. 254 for DFA), its prediction performance is unsatisfactory. That is, LASSO incorrectly ignores many informative features. To further evaluate whether the improvement of DFA is significant or not, we further calculate the *p*-values between DFA and other strategies. We first check the considered data is normal or not. If it is normal, we use *t*-test. Otherwise, we use wilcoxon rank test (Taheri and Hesamian, 2013). The *p*-values indicate that, the performance of DFA is statistically outperform other methods.

**Table 4:** Comparison of different strategies of feature selection on benchmark training datasets.

Strategy		Sensitivity	Specificity	Precision	Accuracy	MCC	F1	AUC	Number of features
Combination of all features	Average stdev *p*-value	0.707 ± 0.017 6.9e-10	0.716 ± 0.010 5.2e-10	0.713 ± 0.008 5.6e-11	0.711 ± 0.009 1.4e-11	0.423 ± 0.018 1.4e-11	0.710 ± 0.011 2.4e-11	0.765 ± 0.010 4.7e-19	470 N/A N/A
LASSO	Average stdev *p*-value	0.734 ± 0.016 5.6e-05	0.750 ± 0.004 1.6e-05	0.746 ± 0.006 2.2e-06	0.742 ± 0.009 1.2e-06	0.484 ± 0.017 1.1e-06	0.740 ± 0.011 2.2e-06	0.784 ± 0.006 2.3e-18	74 ± 11 1.8e-20
GA	Average stdev *p*-value	0.752 ± 0.024 0.16	0.755 ± 0.009 5.2e-04	0.754 ± 0.009 4.8e-04	0.753 ± 0.013 3.3e-03	0.507 ± 0.025 3.2e-03	0.753 ±0.016 0.01	0.813 ± 0.006 9.4e-16	233 ± 11 3.2e-05
DPSO	Average stdev *p*-value	0.752 ± 0.013 0.0248	0.752 ± 0.005 4.7e-05	0.753 ± 0.006 5.7e-05	0.751 ± 0.008 1.7e-04	0.504 ± 0.016 1.7e-04	0.752 ±0.009 4.7e-04	0.819 ± 0.004 1.4e-16	280 ± 6 5.8e-10
DFA	Average stdev *p*-value	0.763 ± 0.007 N/A	0.777 ± 0.014 N/A	0.774 ± 0.012 N/A	0.770 ± 0.009 N/A	0.540 ± 0.018 N/A	0.768 ±0.008 N/A	0.876 ± 0.005 N/A	254 ± 4 N/A

*The threshold is set where the MCC achieve the maximum value. LASSO, DFA, DPSO, and GA stand for least absolute selection and shrinkage operator, discrete firefly algorithm, discrete particle swam optimization, and genetic algorithm, respectively.*

### Comparison With Other Predictors on Benchmark Testing Datasets

We compare our method with SecretomeP, SREpred, iMSP on benchmark testing dataset. Table 5 reports the average prediction performance of the considered predictors. SecretomeP and SRTpred output the average AUC values of 0.709 and 0.714, and achieve the average MCC values of 0.426 and 0.431. General and species-specific models of iMSP produce slightly different results (0.449 vs. 0.469 for MCC values, and 0.795 vs. 0.817 for AUC values). Our method yields decent performance with the average MCC of 0.519 (∼11% higher than the second best iMSP-*H*) and the average AUC of 0.844 (∼4% higher than the second best iMSP-*H*). It also gives out the best sensitivity and specificity among all considered methods. Particularly, the calculated *p*-values indicates the improvements of SCRIP compared with other predictors are statistically significant.

**Table 5:** Comparison of SCRIP with other state-of-the-art predictors on benchmark testing datasets.

Predictor		Sensitivity	Specificity	Precision	Accuracy	MCC	F1	AUC
SecretomeP	Average stdev *p*-value	0.700 ±0.025 l.1e-08	0.726 ± 0.033 1.0e-06	0.719 ± 0.025 4.5e-08	0.713 ± 0.021 4.4e-09	0.426 ± 0.042 4.4e-09	0.709 ± 0.020 2.5e-09	0.709 ± 0.011 9.6e-20
SRTpred	Average stdev *p*-value	0.710 ± 0.021 1.0e-07	0.721 ± 0.026 2.8e-05	0.718 ± 0.017 2.0e-06	0.715 ± 0.012 2.1e-07	0.431 ± 0.024 2.2e-07	0.714 ± 0.012 9.1e-08	0.714 ± 0.018 1.0e-14
iMSP-*U*	Average stdev *p*-value	0.718 ± 0.031 1.2e-07	0.730 ± 0.026 6.6e-05	0.727 ± 0.026 8.3e-06	0.724 ± 0.026 1.2e-06	0.449 ± 0.052 1.2e-06	0.723 ± 0.027 4.3e-07	0.795 ± 0.009 4.4e-13
iMSP-*H*	Average stdev *p*-value	0.733 ± 0.027 3.9e-06	0.735 ± 0.025 1.4e-04	0.735 ± 0.019 3.5e-05	0.734 ± 0.018 8.9e-06	0.469 ± 0.036 9.2e-06	0.734 ± 0.019 5.1e-06	0.817 ± 0.012 1.4e-10
SCRIP	Average stdev *p*-value	0.754 ± 0.027 N/A	0.765 ± 0.036 N/A	0.763 ± 0.029 N/A	0.759 ± 0.024 N/A	0.519 ± 0.047 N/A	0.758 ± 0.023 N/A	0.844 ± 0.010 N/A

*The threshold is set where the MCC achieve the maximum value.*

### Comparison With Other Predictors on iMSP’s Testing Dataset

We test the predictive performance of the proposed method on iMSP’s testing dataset. It contains 398 secretory proteins and 2126 non-secretory proteins. We compare our method with SecretomeP, SREpred, and iMSP. We use the general model (iMSP-*U*) and species-specific model (iMSP-*H*) of iMSP. The results are listed in Table 6. Comparatively, the proposed method produces a good result with the sensitivity of 0.716 and the specificity of 0.884. Although our specificity is not the highest, it is slightly lower than the highest specificity of iMSP-*H*. The latter gives out the specificity of 0.908. However, we yield much higher sensitivity at 0.716 than that of 0.538 for iMSP-*H*. We achieve the highest MCC and AUC values of 0.537 and 0.845, respectively. They are about 22% (0.537/0.443≈1.22) and 5% (0.865/0.821≈ 1.05) higher than the second best iMSP-*U*. It proves our predictor has a good performance of generalization.

**Table 6:** Comparison of stat-of-the-art predictors with the proposed method on iMSP’s testing dataset.

Predictor	Sensitivity	Specificity	Accuracy	MCC	AUC
SecretomeP	0.632	0.787	0.762	0.340	0.764
SRTpred	0.678	0.802	0.782	0.392	0.770
iMSP-*U*	0.631	0.866	0.829	0.443	0.821
iMSP-*H*	0.538	0.908	0.850	0.441	0.817
SCRIP	0.716	0.884	0.858	0.537	0.865

### Application to Cancer Biomarkers Identification

In this research, we adopt the proposed method to recognize cancer biomarkers. To do this, we collect four sets of cancer proteins from the Human Protein Atlas (Uhlen et al., 2010). We collect 2,451 breast cancer proteins, 257 gastric cancer proteins, 2,838 lung cancer proteins, and 317 pancreatic cancer proteins. Then we remove the proteins with less than 50 residues. Next, we map these proteins into Swiss-Prot to extract secretory proteins. After that, we map the secretory proteins into the Human Plasma Proteome Project to obtain related secretory plasma proteins. We finally obtain 52, 15, 60, and 21 secretory proteins in breast cancer, gastric cancer, lung cancer and pancreatic cancer, respectively. We use these proteins as positive samples and the rest proteins as negative samples. Table 7 lists the predictive performance on the considered cancer proteins. Generally, SCRIP produces a decent prediction of cancer biomarkers with the AUC values range from 0.77 to 0.81. However, we notice the relative low values of the MCC and F1 when compared with that on the benchmark training dataset. It is because these datasets are class-imbalanced, which will somewhat influence the threshold-dependent criteria. Actually, SCRIP produces higher than 0.77 of the AUC values on four types of considered cancer proteins.

**Table 7:** Application of SCRIP to cancer biomarkers identification.

Types of Cancer	Sensitivity	Specificity	Precision	Accuracy	MCC	F1	AUC
Breast Cancer	0.769	0.718	0.057	0.719	0.156	0.107	0.776
Gastric Cancer	0.733	0.820	0.193	0.815	0.311	0.306	0.804
Lung Cancer	0.733	0.666	0.045	0.667	0.120	0.085	0.792
Pancreatic Cancer	0.667	0.691	0.135	0.689	0.190	0.224	0.811

## Conclusion

This work proposed a novel computation-based method named SCRIP for the identification of human secretory proteins in plasma/blood. We collected and analyzed a series of sequence-based features, which has been proved to be related to human secretory proteins. These features included relative amino acid composition, secondary structure, evolutionary conservation and physicochemical properties. We used logistic regression, which is fast and less likely to lead to the overfitting, to build the prediction model. In order to get rid of potential redundant features, we introduced discrete firefly algorithm to perform feature selection. The test on benchmark testing datasets and independent testing datasets proves the good generalization of our method. Particularly, we also applied the proposed predictor for the recognition of cancer biomarkers. SCRIP successfully recognized more than 66% of cancer secretory proteins with the AUC values higher than 0.77. We conclude SCRIP is a promising predictor, which relies on novel design and elaborate feature selection strategy, for accurate identification of human secretory proteins in plasma.

## Data Availability

The datasets generated for this study can be found in SCRIP, http://www.inforstation.com/webservers/SCRIP/.

## Author Contributions

JZ conceived the idea of this research, compiled the benchmark datasets, and revised the manuscript critically. YZ performed the research including data collection, test, and analysis. ZM supervised the whole research. All authors have read and approved the final manuscript.

## Conflict of Interest Statement

The authors declare that the research was conducted in the absence of any commercial or financial relationships that could be construed as a potential conflict of interest.
